# Broadband non-reciprocal transmission of sound with invariant frequency

**DOI:** 10.1038/srep19824

**Published:** 2016-01-25

**Authors:** Zhong-ming Gu, Jie Hu, Bin Liang, Xin-ye Zou, Jian-chun Cheng

**Affiliations:** 1 Key Laboratory of Modern Acoustics, MOE, Institute of Acoustics, Department of Physics, Nanjing University, Nanjing 210093, P. R. China; 2 Collaborative Innovation Center of Advanced Microstructures, Nanjing University, Nanjing 210093, P. R. China; 3 Department of Information Science and Technology, Nanjing Forest University, Nanjing, 210037, P. R. China

## Abstract

We design and experimentally demonstrate a broadband yet compact acoustic diode (AD) by using an acoustic nonlinear material and a pair of gain and lossy materials. Due to the capabilities of maintaining the original frequency and high forward transmission while blocking backscattered wave, our design is closer to the desired features of a perfect AD and is promising to play the essential diode-like role in realistic acoustic systems, such as ultrasound imaging, noise control and nondestructive testing. Furthermore, our design enables improving the sensitivity and the robustness of device simultaneously by tailoring an individual structural parameter. We envision our design will take a significant step towards the realization of applicable acoustic one-way devices, and inspire the research of non-reciprocal wave manipulation in other fields.

One-way manipulation of acoustic waves is highly desirable in a great variety of scenarios, but has long been challenging due to the restriction of the well-known reciprocity principle^1^,^2^,^3^,^4^,^5^. Our realization of an “acoustic diode” (AD) has for the first time broken through this barrier and enabled rectification of acoustic waves by coupling a nonlinear medium with acoustic superlattices^1^,^2^. Recently, Alù and colleagues have realized acoustic isolation by using external fluid flows to break space-time symmetry^3^. Popa *et al.* have proposed an active acoustic metamaterials to achieve unidirectional transmission with compact structure^4^. As nonlinear optical isolator is a hot topic of broad interests^6^,^7^,^8^, these pioneering works of ADs has attracted many attentions which may offer the potential to revolutionize the existing acoustic techniques in different important fields. In reality, however, this expectation is far beyond reach in respect that in such ADs the transmitted acoustic wave along the “positive” direction has shifted frequency or attenuated amplitude as compared to the incident wave. As a result, such AD prototypes cannot be coupled with other acoustic devices and play the crucial role like its electrical counterpart does in electrical circuits. The emergence of nonlinear ADs has also been followed by considerable efforts dedicated to the pursuit of linear acoustic one-way devices^9^,^10^,^11^,^12^. Despite the significantly improved performances of the resulting linear devices including high efficiency, broad bandwidth, and, esp. invariant frequency during transmission, they cannot be regarded as practical ADs since the reciprocity principle still holds in such systems due to their linear nature^13^. In other words, the one-way effect can only be realized for incident wave with particular wavefront incident along particular directions, e.g., normally incident plane wave from two opposite sides. So far, a perfect AD with the potential to really revolutionize the current techniques used in current practical applications, as we have once expected such kinds of devices to do, still remains challenging.

In this article, we have made a theoretical and experimental attempt to address this difficulty. Our proposed model has a sandwich structure that consists of a pair of acoustic gain and lossy media and another acoustical nonlinear material inserted in between. The introduction of gain and lossy effects enables asymmetric manipulation of amplitudes for incident waves from two opposite directions. On other hand, we employ the nonlinear material to provide pressure-dependent response to the incident wave, apart from its fundamental necessity for breaking the reciprocal principle^14^. Theoretical analysis reveals that by suitably tuning the structural parameters, the resulting devices, formed by coupling the nonlinear material with the gain/loss pair, could therefore fulfill the functionality a perfect AD is expected to possess that allows acoustic waves to pass along a given direction with a near-unity efficiency and invariant frequency while blocking its transmission along the opposite direction. An experimental implementation by active circuits is also presented, and the results verify our scheme and show that the sample device is able to work as predicted in a broad band despite its compact configuration. With the capability of giving rise to non-reciprocal acoustic transmission while preserving the original frequency and the flexibility of further enhancing the sensitivity and robustness simultaneously, our proposed structure may take a significant step towards the realization of applicable acoustic one-way devices with potential applications in many scenarios.

## Results

**Figure 1:**
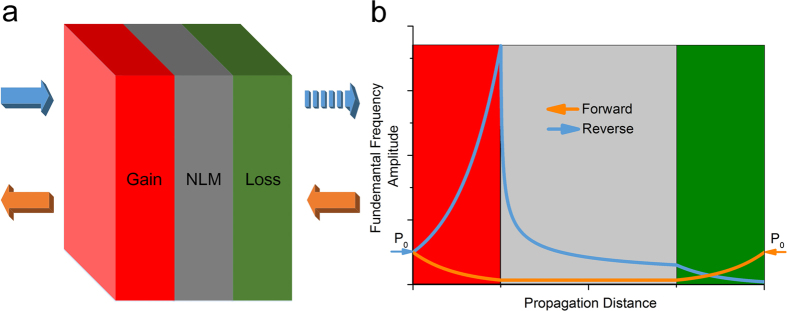
Design and behavior of the acoustic non-reciprocal device with invariant frequency and near-unity forward transmission. (**a**) Schematic of the configuration of the broadband non-reciprocal device. (**b**) The spatial intensity distribution of the waves coming from the two opposite directions, showing the underlying mechanism responsible for the frequency-invariant rectification phenomenon. The forward (Orange arrow) and the reverse (Blue arrow) directions can be generally defined as the propagating directions of the wave incident from the side of loss and gain materials, respectively. The dotted-line arrow indicates that the transmission along this direction virtually vanishes.

Figure 1 schematically shows how the desired non-reciprocal transmission can be realized in the proposed structure comprising an acoustic nonlinear material and a pair of gain and lossy materials with well-tuned complex parameters. The sandwich configuration of our design is displayed in Fig. 1(a). In general, the forward direction of such one-way devices can be defined as the propagating direction of wave incident from the side of the lossy material (from right to left for this particular case, as the orange arrow shows), and the opposite direction can be regarded as the reverse direction (blue arrow). As one of the most important characteristics of nonlinear medium filling the middle region of the whole system, it yields quite different responses when the amplitude of the incident wave varies. When the pressure amplitude of the incident wave is at a sufficiently low level, the nonlinear medium will degenerate to a quasi-linear system and will respond linearly to such a small-amplitude wave. If the viscosity effect is negligible in this medium, the incident wave can pass through it without any change in the amplitude or frequency. As we gradually increase the strength of the input signal, however, the medium will exhibit stronger and stronger nonlinearity effect, characterized by the enhancement of the amount of acoustic energy transferred into second and higher harmonic waves, which implies that the transmission of the remaining fundamental wave is amplitude-dependent and will decrease as the input acoustic wave propagates. This offers the possibility to give rise to asymmetric transmissions for acoustic wave incident from two opposite sides if the amplitudes of signals from left and right sides can be harnessed differently via controlled amplification and attenuation processes. Equivalently, the manipulation of pressure amplitude can be realized by tailoring the effective refractive index in the complex domain. The recent advance in acoustic metamaterials has extended the concept of acoustic manipulation from passive control^15^,^16^,^17^,^18^,^19^ to active control^4^,^20^,^21^,^22^. For a plane acoustic wave propagating in a homogeneous medium, the acoustic pressure can be expressed as: 
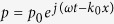
. Here, 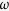 and 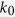 are the angular frequency and the wave number in the background medium, respectively. For material with a complex refractive index 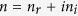, this relationship can be rewritten as 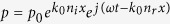. If the imaginary part of effective refractive index is positive (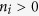), the amplitude of acoustic waves will be exponentially amplified as the propagating distance increases, and this kind of material can be referred to as a gain medium whose practical implementation will be discussed later. For the so-called lossy medium characterized by an effective refractive index with a negative imaginary part (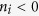), the amplitude of acoustic waves will undergo an exponential attenuation as it propagates, and this kind of material can be marked as lossy medium. When we couple the nonlinear medium with a pair of gain and lossy medium as shown in Fig. 1(a), an asymmetric transmission can be expected since the wave incident from the two sides will enter the nonlinear medium with quite different pressure amplitudes.

A large gap still needs to be filled, however, between such an asymmetric transmission and the desired one-way effect. For the purpose of achieving a perfect non-reciprocal transmission for which the forward transmitted waves will keep the original amplitude and frequency characteristics, it is vital to delicately tailor the structural parameters in the system to meet two crucial requirements: the gain and loss must be sufficiently high and, meanwhile, satisfy the complementary condition^7^. For acquiring a clearer insight into the underlying physics in our design, we will analyze the behaviors of the incident wave travelling along the forward and the reverse directions respectively, which can be predicted qualitatively by the results in Fig. 1(b) displaying the spatial distributions of the acoustic energy within the structure for these two particular cases. The subscripts G and L represent the gain and lossy media in the following discussion, respectively. When the gain and loss of the system are high enough and in balance, the acoustic waves incident from the right side impinge on the lossy medium first within which the amplitude of the incident waves will be attenuated to a considerably low value and can be expressed as 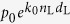. Due to the quasi-linear nature of the nonlinear medium in this case, the wave will leave this region with preserved frequency and attenuated amplitude. After that, the wave will enter the gain materials where the amplitude can be amplified to 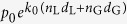, which is restored to the original value because the whole structure has a balanced gain and loss, i.e., 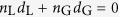, as shown by the orange line in Fig. 1(b). However, when the acoustic wave comes from the reverse side, as the blue line in Fig. 1(b) indicates, it will enter the gain medium first and then leave it with amplitude amplified to a remarkably high value 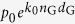, which means the nonlinear conversion from the fundamental waves to high orders harmonic waves will be extremely strong within the nonlinear medium. This obviously results in significantly diminished amplitude of the fundamental wave and the value of 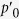 will be much smaller than 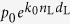. When the resulting wave passes through the lossy medium, the amplitude of the fundamental wave will undergo a second decrease, which can be expressed as 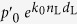. As a result, the total transmission along this direction may become negligible if the conversion rate and loss are high enough. This can be understood as the essential mechanism of the designed non-reciprocal device. Here, we should point out that even if there exist reflections between different layers, we can adjust the amplification factor dynamically to meet the balanced condition.

**Figure 2:**
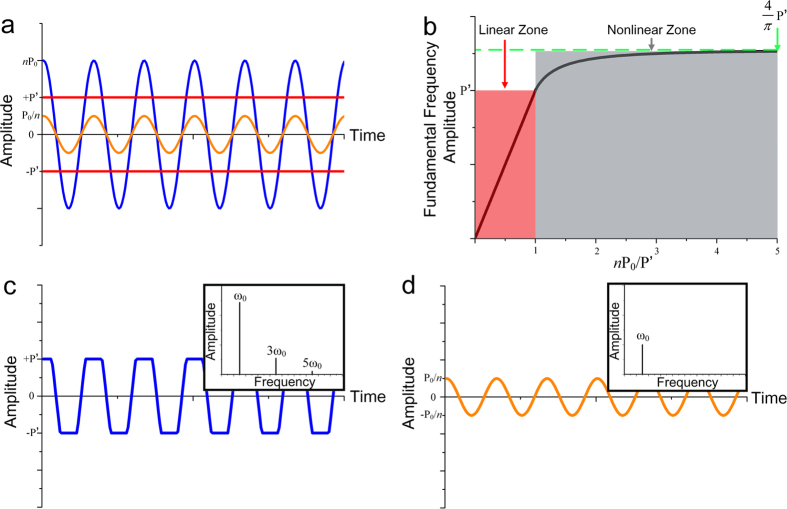
Time-domain and frequency-domain waveforms of the input and output signals of the nonlinear medium. (**a**) Time-domain input waveform along the forward and reverse directions after passing through the lossy and gain media they meet first. (**b**) The amplitude dependence of the nonlinear medium. (**c,d**) show the time-domain signal after passing through the nonlinear medium along the reverse and forward directions respectively. The insets are the corresponding spectrum components. Along the reverse direction, the wave has its peak clipped off when passing through the nonlinear medium, and the amplitude of transmitted fundamental wave is then largely diminished. The wave travelling along the positive direction is not affected by the nonlinear medium due to its small amplitude.

For a clear physics picture, we will inspect the time-domain waveform of the wave entering and leaving the nonlinear medium, which is the key part providing pressure-dependent property crucial for achieving the nonreciprocal transmission, and analyze the variation in the amplitude of spectrum components as the wave travels through this part along the two opposite directions. As a typical nonlinear circuit, a limiter circuit allows signals below a specified input power to pass unaffected while attenuating the peaks of stronger signals exceeding this input power. Here, we assume the nonlinear medium have the property of limiter circuit that has a ‘turn on’ value to distinguish the linear and nonlinear zones. Then, we will have three typical signals in this system, as shown in Fig. 2(a). The blue (orange) sinusoid represents the amplified (attenuated) wave which can be regarded as the output signal of the gain (lossy) material and will pass through the nonlinear medium along the reverse (forward) direction. And the red line represents the specified value of the limiter, which keeps a constant over time. Here,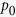, 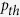 and 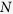 are the original amplitude of the incident waves, the threshold value and the amplification factor. With the changing of the amplification factor from small to large, it is obvious that the nonlinear part will have a different response to the incident waves, as shown in Fig. 2(b). At first, the amplification factor is very small and the amplified value is smaller than the threshold value, then the limiter will work on the linear zone, as depicted as the red zone, which means the transmitted waves will have the same frequency characteristic as the incident waves. However, when the amplified value exceeds the specified value, the limiter will have a nonlinear response, as depicted as the grey zone. The high orders harmonic waves will be generated, and the amplitude of the fundamental wave will approach saturation. Besides, when the amplified value is large enough, the distorted sinusoid waves will be a very close approximation to the square waves, which means the saturation point 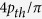 can be predicted by the Fourier transform of the square waves, as depicted as green dashed lines. Apparently, in this design, it is desirable that the amplified amplitude is much larger than the specified value of the limiter while the attenuated amplitude is quite below it. Then the nonlinear part can be used to modulate the acoustic wave incident from the two sides to create the non-reciprocal transmission. Figure 2(c) shows the amplified waves modulated by the limiter in time domain and the inset is the frequency spectrum. It is clear that along the reverse direction, the wave has to have its peak clipped off when passing through the nonlinear medium, which leads to the generation of the high orders harmonic waves and significant reduction in the amplitude of transmitted fundamental wave. The wave will then enter the lossy medium to undergo the second attenuation. On the other hand, Fig. 2(d) shows the time-domain waveform of the attenuated waves after passing through the limiter, and the inset is the corresponding spectrum components. The wave travelling along the forward direction is not affected by the nonlinear medium due to the fact that the amplitude of such attenuated waves is lower than the turn-on value. Hence the limiter works in the linear zone, which means the transmitted wave will have an invariant frequency. The wave along this direction will then have its amplitude restored to the initial value after propagating through the gain medium.

**Figure 3:**
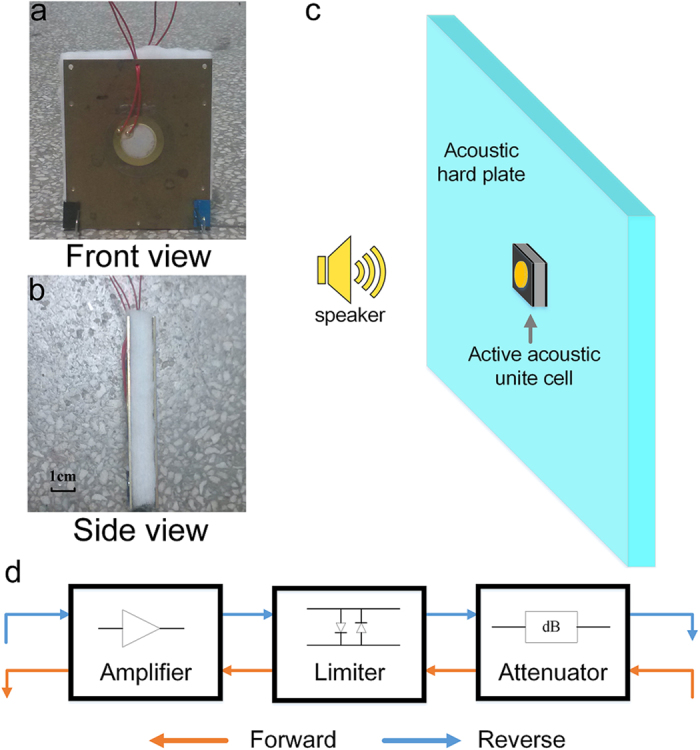
Experimental set-up. Photograph of basic unit cell is shown in (**a**) (Front view) and (**b**) (Side view). (**c**) Schematic of the experimental environment. (**d**) The block diagram of nonlinear electronic circuit.

For verifying the effectiveness of our scheme, we have fabricated a prototype of the designed device and measured its non-reciprocal performance experimentally. For natural acoustic materials, the refractive index should have a negative imaginary part, indicating that the material is lossy with inherent damping. In contrary, acoustic gain materials with a positive imaginary part of the effective refractive index are usually unavailable in nature, which have recently attracted considerable attentions^20^,^21^,^23^,^24^. Cummer and coworkers have achieved the acoustic gain effect by using active acoustic unite cell, which has inspired many potential applications^4^,^23^. Compared to the conventional acoustic nonlinear medium, on the other hand, the nonlinear behavior generated by the electronic circuit will be more robust. Therefore we employ electronic circuits in the current study as a specific implementation. But, compared to the previous acoustic devices (e.g., active noise canceling headphones) that go beyond the restriction of reciprocity by using active elements, our design aims to achieve nonreciprocal transmission with original amplitude and frequency. Besides, we need to emphasize, however, this is only for the sake of an unambiguous demonstration of the effectiveness of our design that is in fact general and may be implemented by any practical acoustical systems satisfying the above-mentioned requirements on the structural parameters. The front view and side view of the fabricated unit cell have been shown in Fig. 3(a,b). The thickness of the device is 2.2 cm, which is only 1/5 wavelength since the working frequency we chose is 3 kHz. As seen in the figures, the unit cell has a symmetry structure composed of five layers. For the sake of unambiguously identifying the amount of the transmitted acoustic energy, we fill the middle region of the structure with sound-absorbing foam to absorb the unwanted transmitted waves and isolate the vibration, and couple the sound-absorbing layer to two brass plates, which can acoustically treated as a rigid boundary for airborne sound, to block the waves. Two 35-mm-diameter piezoelectric (PZT) ceramic films, which have been widely used in the previous designs of acoustic active unit cell and proven to be quite good candidate for acoustic gain material, have been attached to the two brass plates. Here, we employ it as sensing and driving transducers since it has the ability of reciprocal conversion between acoustic energy and electric energy^25^. The experiment set-up has been shown in Fig. 3(c). The unit cell we designed has been fixed in the middle of an acoustic hard plate which is used here to avoid the unnecessary diffraction. The incident wave is excited by a speaker in one side of the plate and the transmitted signal is measured in the other side. Two PZT ceramic films are connected to the electronic circuit whose block diagram is presented in Fig. 3(d). The amplifier circuit is based on the design of LM386 audio amplifier and has an amplification of 20^26^. The limiter circuit mainly consists of an antiparallel diode pair. And some dissipative conductors are arranged to implement the attenuator part. Furthermore, this device can be conveniently tuned to work for incident waves with different amplitudes by simply adjusting the parameters in the circuit.

**Figure 4:**
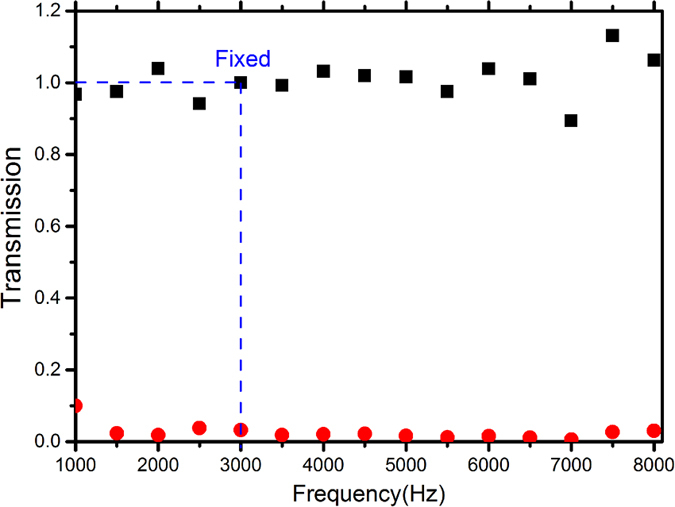
The transmission measurements. The experimental results of the transmissions along the forward (black squares) and reverse directions (red dots) are plotted for comparison. The blue dashed line indicates the location of the working point, chosen as 3 kHz in the current design, for which the resulting device is desired to yield the optimal performance. The measurements were repeated 3 times and the results are virtually identical each time.

We have measured the performance of the prototype within a broad frequency band ranging from 1 kHz to 8 kHz. The speaker is controlled by a signal generator and launches sinusoid signal for which the frequency is swept with a step of 500 Hz. To characterize the non-reciprocal transmission efficiency, we measure the amplitude of the incident signals and the transmitted signals along the two opposite directions, and evaluate the discrepancy between the transmissions of forward and reverse waves. For a quantitative estimation, we define the transmission along the forward direction as 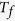 and the transmission along the reverse direction as 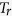 respectively. The measurement results have been plotted in Fig. 4 in which 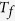 and 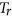 are represented by black square scatter and red circle scatter, respectively. In the measurement, we have adjusted the system parameters delicately to guarantee an optimal performance of the device at a particular frequency, chosen as 3 kHz in the current design, which means that the forward transmission is virtually unity as the driving frequency is exactly at this frequency. When the incident wave of the same frequency travels along the reverse direction, the transmission will be significantly diminished, as predicted by the fundamental mechanism of the structure we propose. It is noteworthy, however, although the working point is set at this individual frequency, non-reciprocal transmission property of the prototype persists within an ultra-broad band except for slight fluctuations of the values of the forward transmissions around 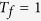 which may be caused by the dynamic impedance mismatch in the system.

## Discussion

Realization of a perfect AD has long been challenging, to which considerable efforts have been and continue to be devoted. By exploiting the characteristic of nonlinear medium that it has a nonlinear response to the input signals with different amplitude, our proposed structure consists of acoustic nonlinear medium, acoustic gain and lossy materials to realize the nonreciprocal transmission. Compared the previous designs, the transmitted waves have an invariant frequency exactly the same as the incident waves and the amplitude can be tuned to unity, which are very close to what a perfect AD is expected to offer. Furthermore, it is observable from Fig. 2 that by adjusting the parameters of the nonlinear medium, we can further reduce its turn-on value representing the transition from the linear regime to the nonlinear regime, which may endow the resulting device with an intriguing feature. By reducing this individual parameter, it is possible to decrease the threshold value for the amplitude of incident wave to “conduct” along the forward direction and, meanwhile, make it harder for the structure to allow the reverse wave with extremely large amplitude to pass in a similar manner to the breakdown effect in electrical diodes. In other words, this offers the potential to enhancing the sensitivity and robustness of our one-way device simultaneously, which is of paramount significance for the application of such devices in practice. Consequently, we anticipate our design, with the capability of realizing broadband non-reciprocal transmission and the flexibility of being tailored to further improving the performance, will make a significant step forward in the pursuit of a perfect AD and have potential applications in a great variety of scenarios.

## Additional Information

**How to cite this article**: Gu, Z.-m. *et al.* Broadband non-reciprocal transmission of sound with invariant frequency. *Sci. Rep.* **6**, 19824; doi: 10.1038/srep19824 (2016).
